# A176 RESTORATION OF IMPAIRED MICROBIOTA-MEDIATED ARYL HYDROCARBON RECEPTOR SIGNALING IN PATIENTS WITH CELIAC DISEASE BY ORAL TRYPTOPHAN SUPPLEMENTATION: AN EXPLORATORY STUDY

**DOI:** 10.1093/jcag/gwad061.176

**Published:** 2024-02-14

**Authors:** U Kirtikar, G H Rueda, J Szeto, H Galipeau, M Constante, X Wang, M Pinto-Sanchez, D Armstrong, P Bercik, E Verdu

**Affiliations:** Medicine, McMaster University, Hamilton, ON, Canada; Medicine, McMaster University, Hamilton, ON, Canada; Medicine, McMaster University, Hamilton, ON, Canada; Medicine, McMaster University, Hamilton, ON, Canada; Medicine, McMaster University, Hamilton, ON, Canada; Medicine, McMaster University, Hamilton, ON, Canada; Medicine, McMaster University, Hamilton, ON, Canada; Medicine, McMaster University Faculty of Health Sciences, Hamilton, ON, Canada; Medicine, McMaster University Faculty of Health Sciences, Hamilton, ON, Canada; Medicine, McMaster University Faculty of Health Sciences, Hamilton, ON, Canada

## Abstract

**Background:**

Celiac disease (CeD) is an autoimmune condition driven by gluten in individuals expressing celiac-specific genes (HLA-DQ2 and/or DQ8). A strict gluten-free diet (GFD) is currently the only treatment. 30-40% of celiac patients exhibit persistent symptoms despite following a GFD ampersand:003E1 year and are considered non-responsive. Tryptophan is an essential amino acid metabolized by gut microbiota to produce indoles that activate aryl hydrocarbon receptor (AhR), contributing to intestinal barrier homeostasis. Our previous study showed CeD patients have altered tryptophan metabolism and limited AhR activation, only partially improving with a GFD. In healthy volunteers, oral tryptophan supplementation improved AhR activation in the small intestine, thus, indicating its feasibility as adjuvant to the GFD in non-responsive CeD.

**Aims:**

The study aims were to: 1) determine whether tryptophan supplementation improves celiac specific symptoms in non-responders; and 2) if tryptophan supplementation modifies gut microbiota composition, intestinal indole production and AhR activity.

**Methods:**

The study is a double-blind, randomized, placebo-controlled trial with the aim to recruit 50 adults from the McMaster University Celiac Clinic. At visit 1, consented participants are randomized 1:1 to L-tryptophan (3g/day) or placebo and receive intervention capsules to be taken for 3 weeks after Visit 2. At Visits 2 and 3, participants complete dietary and symptom questionnaires, provide blood, stool, and urine samples, and undergo endoscopy. The primary outcome, improvement in symptoms, is assessed using the Celiac Symptoms Index (CSI); general GI symptoms by Gastrointestinal Symptoms Rating Scale (GSRS); mood symptoms by Hospital Anxiety and Depression Scale (HADS); quality of life by Patient Assessment of Upper Gastrointestinal Disorders-Quality of Life questionnaire (PAGI-QoL).

**Results:**

To- date we have recruited 10 participants. We will present blinded results from participants that have completed the study at the time of presentation based on responses to questionnaires pre, and post tryptophan/placebo intervention. At the time of analysis of this abstract, 5 participants had completed the study showing reduced CSI scores post intervention. The GSRS was also significantly reduced post intervention however, no change was observed in HADS and PAGI-QoL.

**Conclusions:**

As all these results are currently blinded, we cannot attribute the symptom improvement to either tryptophan or the placebo effect. After unblinding the data, we will analyze the data to determine the extent of symptom reduction in relation to tryptophan metabolites (from urine, stool and duodenal aspirates) and AhR activation (from duodenal biopsy samples).

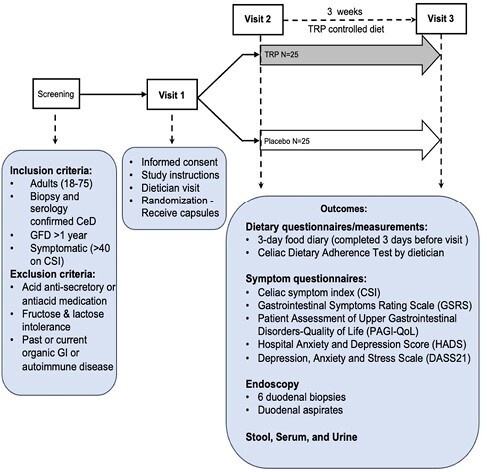

**Funding Agencies:**

Weston Family Foundation

